# Combination immunotherapy induces post-intervention control of HIV

**DOI:** 10.21203/rs.3.rs-6141479/v1

**Published:** 2025-03-19

**Authors:** M.J Peluso, D.A Sandel, A.N Deitchman, S.J Kim, T Dalhuisen, H.P Tummala, R Tibúrcio, L Zemelko, G.M Borgo, S.S Singh, K Schwartz, M Deswal, M.C Williams, R Hoh, M Shimoda, S Narpala, L Serebryannyy, M Khalili, E Vendrame, D SenGupta, L. S Whitmore, J Tisoncik-Go, M Gale, R.A Koup, J.I Mullins, B.K Felber, G.N Pavlakis, J.D Reeves, C.J Petropoulos, D.V Glidden, M.H Spitzer, L Gama, M Caskey, M.C Nussenzweig, K.W Chew, T.J Henrich, S.A Yukl, L.B Cohn, S.G Deeks, R.L Rutishauser

**Affiliations:** 1 Department of Medicine, University of California, San Francisco, San Francisco, CA, USA; 2 Department of Otolaryngology-Head and Neck Surgery, University of California, San Francisco, San Francisco, CA, USA; 3 Department of Microbiology and Immunology, University of California, San Francisco, San Francisco, CA, USA; 4 Department of Clinical Pharmacy, University of California, San Francisco, San Francisco, CA, USA; 5 Department of Medicine, San Francisco Veterans Affairs Medical Center, San Francisco, CA, USA; 6 Vaccine Research Center, National Institute of Allergy and Infectious Diseases, National Institutes of Health, Bethesda, MD, USA; 7 Gilead Sciences, Inc., Foster City, CA, USA; 8 Department of Immunology, Center for Innate Immunity and Immune Disease, School of Medicine, University of Washington, Seattle, WA, USA; 9 Current affiliation: Department of Microbiology and Immunology, and the Institute on Infectious Diseases, University of Minnesota, Minneapolis, MN, USA; 10 Department of Microbiology, University of Washington, Seattle, WA, USA; 11 Basic Research Laboratory, Center for Cancer Research, National Cancer Institute, Frederick, MD, USA; 12 Labcorp-Monogram Biosciences, South San Francisco, CA, USA; 13 Department of Epidemiology and Biostatistics, University of California, San Francisco, CA, USA; 14 Helen Diller Family Comprehensive Cancer Center, University of California, San Francisco, San Francisco, CA, USA; 15 Parker Institute for Cancer Immunotherapy, University of California, San Francisco, San Francisco, CA, USA; 16 Current affiliation: Instituto Butantan, São Paulo, Brazil; 17 Department of Clinical Investigation, The Rockefeller University, New York, NY, USA; 18 Laboratory of Molecular Immunology, The Rockefeller University, New York, NY, USA; 19 Howard Hughes Medical Institute, Chevy Chase, MD, USA; 20 Department of Medicine, University of California, Los Angeles, Los Angeles, CA, USA; 21 Fred Hutchinson Cancer Center, Seattle, WA, USA

## Abstract

The identification of therapeutic strategies to induce sustained antiretroviral therapy (ART)-free control of HIV infection is a major priority.^1^ Combination immunotherapy including HIV vaccination, immune stimulation/latency reversal, and passive transfer of broadly neutralizing antibodies (bNAbs) has shown promise in non-human primate models,^2–7^ but few studies have translated such approaches into people. Here, we performed a single-arm, proof-of-concept combination study of these three approaches in ten people with HIV on ART that included (1) therapeutic vaccination with an HIV/Gag conserved element (CE)-targeted DNA+IL-12 prime/MVA boost regimen followed by (2) administration of two bNAbs (10–1074 and VRC07–523LS) and a toll-like receptor 9 (TLR9) agonist (lefitolimod) during ART suppression, followed by (3) repeat bNAb administration at the time of ART interruption. Seven of the ten participants exhibited partial (low viral load set point) or complete (aviremic) post-intervention control after stopping ART, independent of residual bNAb plasma levels. Robust expansion of activated CD8+ T cells early in response to rebounding virus correlated with lower viral load set points. These data suggest that combination immunotherapy approaches might prove effective to induce sustained control of HIV by slowing rebound and improving CD8+ T cell responses, and that these approaches should continue to be optimized.

## Introduction

Identifying an intervention that can induce eradication or sustained antiretroviral therapy (ART)-free control of HIV infection is a major biomedical and public health priority.^1^ To date, no single approach has demonstrated a consistent effect in achieving complete (aviremic) or partial (low viral load set point) control in people with HIV (PWH) who discontinue ART.^8^ However, some therapeutic combinations have demonstrated success in non-human primate (NHP) models. For example, in NHPs infected with SIV or SHIV, administration of broadly neutralizing antibodies (bNAbs) alone^2,3^ or in combination with therapeutic vaccination and/or a toll-like receptor (TLR) 7 or IL-15 agonist^4–7^ has been associated with a reduction in the post-ART set point and/or cell-associated viral DNA levels. In these studies, post-intervention viral control consistently correlated with the magnitude, breadth, or function of virus-specific T cells.

Although most therapeutic vaccine studies in PWH failed to alter the post-ART viral load set point, modest effects on viral rebound kinetics after stopping ART have correlated with the magnitude of vaccine-specific T cell responses in some cases.^9–12^ Administration of bNAbs at the time of ART initiation or cessation either alone or in combination with other immunotherapies has been associated with modestly accelerated depletion of the HIV reservoir^13^ and HIV control in a handful of PWH after stopping ART, even after bNAbs have waned.^13–17^ Sustained control of HIV in this setting may be mediated by the formation of immunostimulatory virus:antibody immune complexes that promote antigen presentation and activation of endogenous HIV-specific cytotoxic CD8+ T cells and/or antibodies (termed the “vaccinal effect”).^2,3,16,18–21^ Although the definition of HIV control in these studies was highly variable,^8^ it generally occurred in 10–35% of participants who received interventions; in many cases this proportion was similar to post-treatment control in the placebo arm (<20%). Stricter levels of control (i.e., set points <1,000 copies/mL) were less common.^8^ Across humans and NHPs, consistent trends have emerged: particularly in settings in which the size of the reservoir is small and/or ART was started early in the course of infection, low rates of HIV/SIV control can be observed after stopping ART, and this control may be mediated, at least in part, by an effective virus-specific CD8+ T cell response.^2,3,7,12,14,15^

**Fig. 1.**
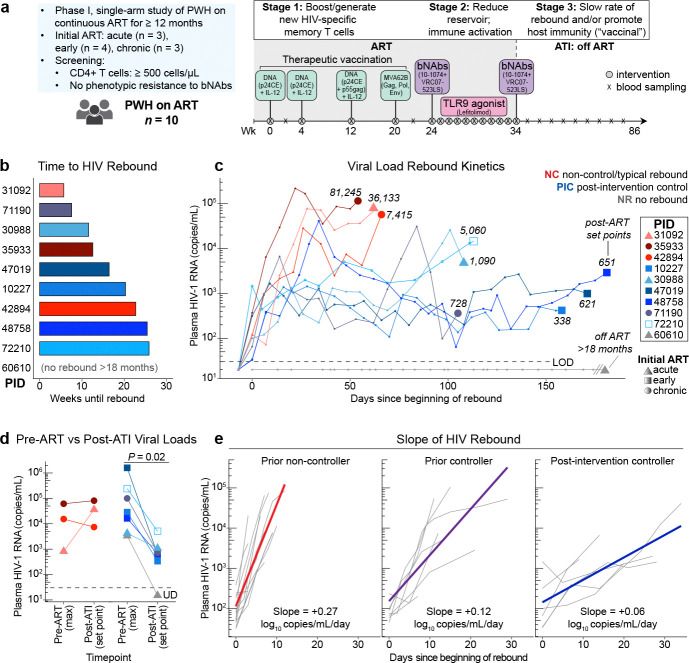
Combination immunotherapy promotes post-intervention control of HIV. a) Study schema: administration of combination immunotherapy including a DNA vaccine targeting conserved elements (CE) of HIV Gag, two broadly neutralizing antibodies (bNAbs) with a toll-like receptor (TLR)9 agonist in 10 people with HIV (PWH) on antiretroviral therapy (ART), followed by a second infusion of bNAbs immediately prior to an analytic treatment interruption (ATI). b) Time to HIV rebound following ART discontinuation. c) Plasma viral load rebound kinetics from the first day of rebound (x=0); numbers on graph indicate set point (median viral load from 2 weeks post-peak viral load to the time of ART re-start); colors indicate rebound phenotype (red, non-control/typical rebound; blue, viremic post-intervention control; grey, no rebound for >18 months off ART); shape indicates timing of ART after HIV acquisition (circle, chronic infection; triangle, acute infection [<1 month]; square, early infection [1–6 months]; end points indicate day of ART restart, except participant with no rebound (grey); empty square indicates participant with HLA-B*57 allele. d) Comparison of maximum recorded viral load prior to ART initiation (single value) versus post-ART set point (Wilcoxon signed rank). e) HIV rebound slope (in log10 copies/mL/day) from time of rebound to time of peak viral load after stopping ART in people who were controllers prior to starting ART (“prior controller,” *n* = 7) or not (“prior non-controller,” *n* = 13) in the absence of any immunotherapeutic intervention, versus the six viremic post-intervention controllers from this trial. MVA, modified vaccinia Ankara. PID, participant ID. NC, non-control. PIC, post-intervention control. NR, no rebound. LOD, limit of detection.

To date, immunotherapy studies in PWH have generally included one or two interventions. In this proof-of-concept study, we sought to determine whether a combination of therapeutic approaches designed to target multiple pathways identified in prior studies might induce virologic control in PWH after discontinuation of ART. Modeled after the NHP studies,^4,5^ we conducted a three stage study in ten PWH on suppressive ART involving (1) therapeutic vaccination (with an HIV Gag conserved-element [CE] DNA/MVA regimen^22–27^) to enhance HIV-specific T cell responses, followed by (2) a combination of two relatively long-acting bNAbs (10–1074^28^ and VRC07–523LS^29^) and a potential latency reversal agent (lefitolimod,^30^ a TLR9 agonist) during ART suppression to reduce the size of the HIV reservoir, and finally (3) administration of the bNAbs immediately prior to stopping ART^31^ and undergoing analytic treatment interruption (ATI) to potentiate host immune responses by slowing rebound and possibly inducing the vaccinal effect (see study schema, Fig. 1a).

## Results

### Study participants

The final study included ten individuals who had initiated ART during the acute, early, or chronic phase of HIV infection (defined as <30 days, between 1–6 months, or ≥6 months following estimated date of infection; *n* = 2, 5, and 3 participants, respectively; see **Extended Data Fig. 1** for CONSORT diagram, **Extended Data Table 1** for a summary of the study population, and Methods for full details). Participants were required to have been consistently suppressed on ART for at least 12 months, to have a screening CD4+ T cell count ≥500 cells/μL and to lack exclusionary comorbidities. We screened HIV reservoir proviruses for susceptibility to the bNAbs and excluded those whose virus was determined to have significantly reduced phenotypic susceptibility to one or both antibodies. The median age was 36 years (range 32–55). The median time since HIV acquisition was 6.7 years (range 1.5–18.2) and the median CD4+ T cell count at enrollment was 736 cells/μL (range 392–1225). The study was conducted during the first three years of the COVID-19 pandemic (2020–2023).

### Safety of combination immunotherapy

Safety events are detailed in **Extended Data Tables 2–4** and **Supplementary Table 1**. Notably, two participants experienced asymptomatic elevations in liver enzymes (one grade 3 event, one grade 4 event) that resolved over 2–3 months without long-term sequelae. Non-study-related events were considered to be the most likely cause of these laboratory abnormalities although a relationship to the study interventions could not be ruled out. These abnormalities resulted in a temporary FDA hold, which resulted in participants receiving a variable number of lefitolimod doses (range 1–9), as well as variability in the time between the first and second bNAb doses (range 10 to 26.4 weeks); one participant did not receive the second infusion of VRC07–523LS due to the above safety concerns. See **Supplementary Table 2** for participant-level demographic information, clinical history, and dosing schedule.

### Effect of the interventions on HIV rebound kinetics

The median length of the ATI was 36.7 weeks (range 14.7–77.1). Plasma HIV RNA rebound, defined as the first of two consecutive quantifiable (>30 copies/mL) plasma HIV-1 RNA levels, occurred at a median of 16.4 weeks (range 5.7–25.9) after ART interruption (Fig. 1b). Prior to rebound, three participants experienced 2–4 weeks in which plasma HIV RNA was intermittently detectable but not high enough to be quantifiable with standard assays (see **Supplementary Table 3** for full viral load data). Strikingly, seven of the ten participants achieved some degree of post-intervention control, with one aviremic for >18 months off ART (Fig. 1c, grey line) and six with sustained low viral load set points. Two of the low viremic controllers exhibited a slow early rebound pattern (rebound slope 33–58 copies/mL/day; calculated from time of rebound until the highest [peak] viral load within the first six weeks after rebound) and initially maintained viral loads near 1,000–2,000 copies/mL for 2–3 months prior to reaching higher viral loads (Fig. 1c, light blue lines). The other four low viremic controllers exhibited oscillatory viral loads around a low set point <1,000 copies/mL for 3–6 months off ART (Fig. 1c, darker blue lines). The remaining three participants (“non-controllers”; Fig. 1c, red lines) exhibited the typical rapid increase in HIV RNA levels (rebound slope 823–9,462 copies/mL/day) with two having higher viral load set points (36,133 and 81,245 copies/mL) and one having an intermediate set point (7,415 copies/mL). All seven controllers elected to resume ART prior to meeting pre-specified ART restart criteria, often due to external considerations (e.g., COVID-19 diagnosis or the need to receive a COVID-19 or Mpox vaccine). Compared to the available historical maximum plasma viral loads documented prior to starting ART, post-ART viral load set points were similar or higher in the three non-controllers but were significantly lower amongst the seven controllers (median maximum viral load prior to ART of 28,615 copies/mL versus set point after stopping ART of 651 copies/mL; *P* = 0.02; Fig. 1d). Of the six viremic post-intervention controllers, one had initiated ART during chronic infection (participant ID [PID] 71190) and one had an HLA-B*57:01 allele (PID 72210). Plasma antiretroviral drug levels were confirmed undetectable in all participants at multiple timepoints during the ATI (see Methods).

### Slope of HIV rebound in viremic post-intervention controllers

To contextualize the HIV rebound kinetics observed in the six viremic post-intervention controllers from this trial, we compared them to the rebound kinetics of PWH on ART who were enrolled in a separate non-interventional ATI study that was conducted concurrently at the same clinical site (NCT04359186). That study included seven individuals who were known to be spontaneous HIV controllers prior to initiating ART and thirteen who had not exhibited HIV control prior to ART initiation. No intervention was administered, as the study goal was to assess the natural history of viral rebound. Strikingly, the slope of the viral load rebound was significantly lower for both the known prior controllers who interrupted ART in the non-interventional ATI study and the viremic post-intervention controllers from the immunotherapy trial compared to the prior non-controllers from the observational study (estimated population average +0.12 and +0.06, versus +0.27 log_10_ copies/mL/day, respectively; *P* < 0.0001 comparing each of the two controller groups to the prior non-controllers; Fig. 1e). Furthermore, the viremic post-intervention controllers from the immunotherapy trial had significantly slower HIV rebound compared to known prior controllers from the observational study (*P* = 0.0004).

### Relationship between lefitolimod exposure, bNAb pharmacokinetics/susceptibility, and rebound kinetics

**Fig. 2.**
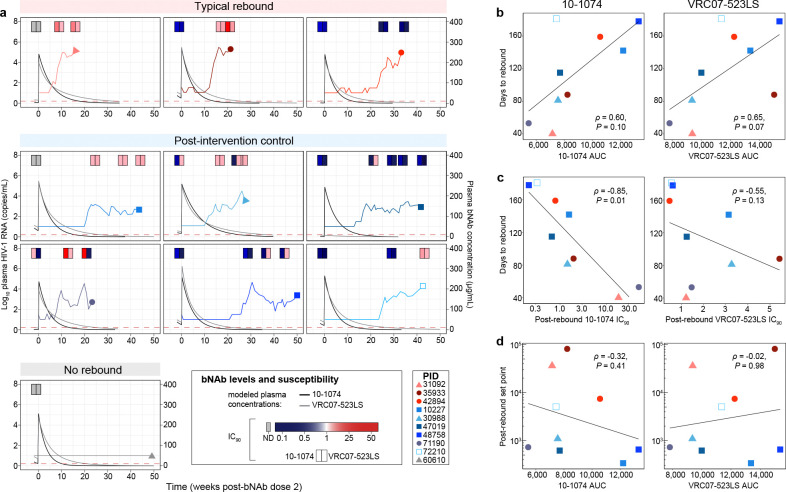
bNAb pharmacokinetics and susceptibility predict time to HIV rebound but not post-ART set point. a) Viral load and modeled bNAb concentrations over time following the second dose of bNAbs in each participant; bNAb line colors: 10–1074, black; VRC07–523LS, grey; at the top of each graph, phenotypic susceptibility of autologous HIV to neutralization by bNAbs (inhibitory concentration [IC_90_]) is depicted in pairs of shaded rectangles (left rectangle: 10–1074; right rectangle: VRC07–523LS); susceptibility was measured from both cell-associated provirus at baseline (depicted in the left-most rectangles in each graph; grey indicates value could not be determined [ND]) as well as from post-rebound plasma virus at multiple timepoints, as indicated. b) Correlation between 10–1074 and VRC07–523LS exposure (AUC, area under the bNAb concentration-time curve following the second dose of bNAbs) and time to rebound. c) Correlation between bNAb susceptibility (IC_90_) post-rebound and time to rebound after ATI. d) Correlation between bNAb exposure and post-rebound viral load set point. Statistical testing: Spearman’s correlation (b-d).

We next sought to understand whether the altered rebound kinetics were related to variation in lefitolimod exposure and bNAb pharmacokinetics (PK). A range of lefitolimod doses were administered (median 8 doses, range 1–9 doses), but the number of lefitolimod doses was not associated with variation in viral load set point (ρ = −0.077, *P* = 0.85). bNAb levels at rebound were variable (0.39–57.6 μg/mL for 10–1074, 0.39–73.4 μg/mL for VRC07–523LS; Fig. 2a). As expected, higher bNAb exposure (area under the curve [AUC] following the second bNAb dose) trended toward being significantly associated with later rebound (10–1074 ρ = 0.60, *P* = 0.10; VRC07–523LS ρ = 0.65, *P* = 0.07, Fig. 2b). Higher phenotypic susceptibility of rebound virus to bNAbs (i.e., lower IC_90_ at the first sample tested after rebound) was significantly associated with later time to rebound for 10–1074 but not VRC07–523LS (10–1074 ρ = −0.85, *P* = 0.01; VRC07–523LS ρ = −0.55, *P* = 0.13, Fig. 2c). In contrast, both bNAb exposure and susceptibility were not linked to differences in post-ART viral load set points (10–1074 AUC ρ = −0.32, *P* = 0.41; VRC07–523LS AUC ρ = −0.02, *P* = 0.98, Fig. 2d; 10–1074 IC_90_ ρ = 0.30, *P* = 0.44; VRC07–523LS IC_90_ ρ = −0.05, *P* = 0.91). Finally, anti-drug antibodies were detected for two participants, although no functional anti-drug antibody impacting bNAb PK was observed (see Supplemental Methods). Therefore, in this study, increased bNAb susceptibility and exposure delayed viral rebound, but could not directly account for the altered post-ART HIV rebound patterns and lower viral load set points.

### Effect of the interventions on the HIV reservoir

**Fig. 3.**
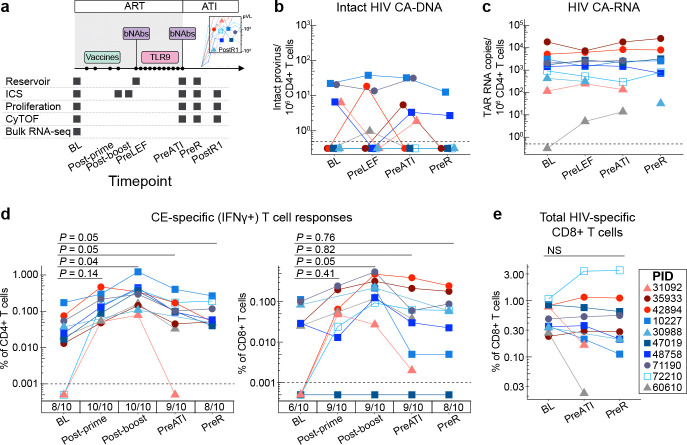
Impact of combination immunotherapy on the HIV reservoir and HIV-specific T cell responses. a) Longitudinal peripheral blood sampling timepoints (applies to Figs. 3–**6**). CD4+ T cell-associated (CA) (b) potentially intact HIV DNA, as measured by IPDA, and (c) HIV RNA (TAR region, indicating total initiated HIV transcripts). d) Magnitude of IFN*γ*+ CE-specific CD4+ and CD8+ T cells as measured by intracellular cytokine staining (ICS); numbers below x-axis indicate the proportion of participants with detectable CE-specific responses at each timepoint. e) Magnitude of total (Gag+Pol+Nef+Env) HIV-specific CD8+ T cell responses by ICS. Statistical testing: Linear mixed effect analysis with Tukey’s multiple comparisons test. IPDA, intact proviral DNA assay. TAR, transactivating response. Timepoints: BL, baseline prior to interventions (i.e., on ART); Post-prime, day of MVA vaccination (>8 weeks after last DNA vaccination); Post-boost, 2 weeks after MVA vaccination; PreLEF, immediately prior to lefitolimod dosing; PreATI, immediately prior to ATI; PreR, last PBMC sampling timepoint available prior to rebound (sampled within 1–4 weeks prior to HIV rebound); PostR1, first PBMC sampling timepoint after rebound (for participants with sample available ≤28 days after rebound, at a low viral load [all <2,600 copies/mL]). IFN*γ*, interferon gamma. NS, no significant change.

The HIV-1 DNA reservoir was measured by digital droplet PCR (IPDA).^32^ In this assay, proviruses are designated as potentially “intact” if the DNA is positive for splice site and envelope sequences. Overall, the participants had low levels of peripheral blood CD4+ T cell-associated intact HIV DNA prior to and during the interventions, mostly fluctuating between 0–10 copies per million CD4+ T cells (see sampling timepoints, Fig. 3a). At baseline, potentially intact HIV DNA was detected in four participants (Fig. 3b), whereas 3’ or 5’ defective HIV DNA was detected in nine participants (**Extended Data Fig. 2a, Supplementary Table 4**). Three viremic post-intervention controllers (PIDs 30988, 47019, and 72210) had no measurable intact HIV DNA at any timepoint. No consistent changes in intact or defective HIV DNA were observed during any of the study stages, although the ability to detect such changes was limited by the low detection frequency and low levels at baseline. While all participants had some CD4+ T cell-associated HIV RNA transcripts detectable in peripheral blood CD4+ T cells at baseline and/or follow up timepoints, these levels were low at baseline, and we did not observe consistent changes over the course of the interventions (Fig. 3c, **Extended Data Fig. 2b**).

There was no obvious relationship between HIV DNA or RNA levels and post-ART viral load set point (see **Supplementary Table 5**). However, the aviremic post-intervention controller (PID 60610) had a unique clinical history and virologic profile. This participant had initiated ART 12 days after acquiring HIV (when the plasma viral load was 3,343 copies/mL) and, in an analysis reported prior to the present trial, replication-competent HIV DNA was not detected in a leukapheresis sample collected 3 years after HIV acquisition, suggesting restricted reservoir seeding during acute infection and very low subsequent HIV burden.^33^ In this trial, the participant had among the lowest levels of peripheral blood cell-associated HIV DNA and RNA, and there was no detectable intact provirus in gut tissue sampled during the ATI period, although there were low levels of detectable non-intact virus (Fig. 3b–c; **Extended Data Fig. 2c**).

### Effect of the interventions on HIV-specific T cell responses

We next explored the relationship between intervention-elicited HIV-specific T cell responses and control. Therapeutic vaccination elicited new or boosted pre-existing interferon (IFN)ɣ+ Gag/CE-specific CD4+ and CD8+ T cell responses in all 10 participants, as measured by intracellular cytokine staining (ICS; median magnitude at baseline prior to the vaccination versus at the peak of the response, 2 weeks after the MVA boost: CD4 0.03% vs 0.341% [p_adj = 0.03]; CD8 0.026% vs 0.158% [p_adj = 0.03]; Fig. 3d; see **Extended Data Fig. 2d** for gating scheme). Gag/CE-specific CD8+ T cells upregulated activation markers (PD-1, CD39) and acquired a more effector-differentiated phenotype in response to the vaccine regimen (i.e., they upregulated T-bet and downregulated CD127; **Extended Data Fig. 2e**). The magnitude of both Gag/CE-specific CD4+ and CD8+ T cell responses contracted back to near baseline levels by the time of the ATI, which occurred a median of 195 days after the MVA boost (range 100–268 days). The T cell boosting effect was specific to vaccine (Gag/CE) antigens, as the vaccine regimen did not significantly alter the frequency or phenotype of HIV Nef-specific T cells (**Extended Data Fig. 2f, g**).

Neither Gag/CE-specific nor total (summed Gag+Pol+Env+Nef) HIV-specific CD4+ or CD8+ T cell responses were significantly boosted during the period of bNAb-mediated viral suppression after ART was stopped (i.e., between PreATI and pre-rebound timepoints; Fig. 3e, **Extended Data Fig. 2h**). Furthermore, there was no correlation between HIV-specific T cell magnitude and post-ART viral load set point (see **Supplementary Table 5**).

### Peri-rebound innate and adaptive immune activation

We next investigated whether broad immune phenotypes and activation, particularly after the ATI, might be associated with the post-ART viral load set point. Because unique virologic mechanisms may have contributed to post-intervention control in the aviremic controller, we focused the following analyses on the six viremic post-intervention controllers.

At baseline, peripheral immune cell type and phenotypic marker frequencies as measured using high-dimensional mass cytometry (CyTOF) as well as broad gene expression patterns as assessed by bulk RNA sequencing of total PBMCs were generally similar between the six viremic post-intervention controllers and the three non-controllers (**Extended Data Fig. 3a,b;** see Fig. 3a for sampling timepoints, **Supplementary Table 6** for CyTOF panels,^34^ and **Extended Data Fig. 3c** for major immune cell type gating and longitudinal frequencies).

**Fig. 4.**
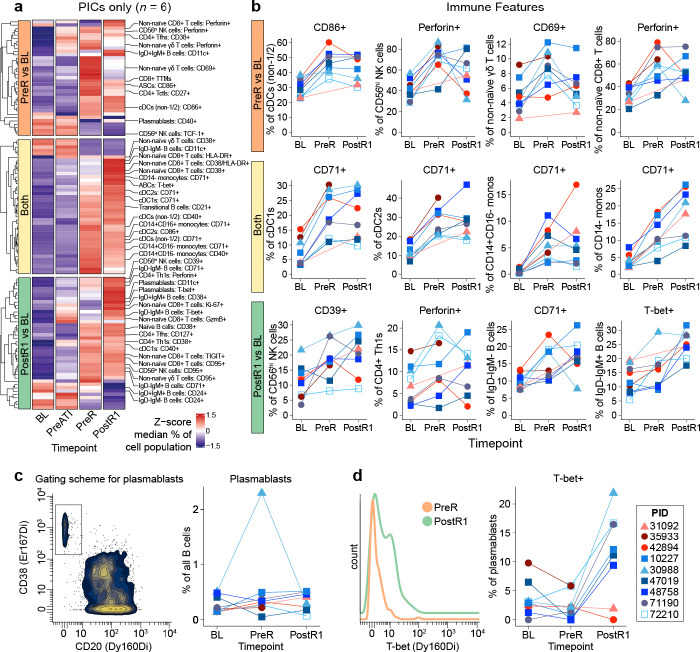
Sequential activation of innate and adaptive immune cells peri-rebound after combination immunotherapy. a) Heatmap depicting the subset of manually gated immune cell features (including cell types and phenotypes) whose abundance changed significantly between baseline compared to pre-rebound (top), baseline compared to the first post-rebound timepoint (bottom), baseline compared to both timepoints (middle) in the six viremic post-intervention controllers, as measured by CyTOF (Wilcoxon signed rank, *P* <0.05; median values are z-scored by feature). b) Participant-level changes in key immune features from (a), also including non-controllers.c ) Gating (left) and longitudinal assessment (right) of total plasmablast abundance. d) Representative plot (left) and longitudinal assessment (right) of the frequency of T-bet+ plasmablasts. See Fig. 3 for timepoint definitions. cDC, conventional dendritic cell. NK, natural killer cell. Th, T helper. Ttm, transitional memory. ASC, antibody secreting cell. ABC, activated B cell. Tctl, cytotoxic CD4+ T cell.

We next asked what changes in broad immune cell types and phenotypes occurred over the course of the trial in the six viremic post-intervention controllers (see **Supplementary Table 4** for full analysis). There were no significant changes in immune cell type frequencies or phenotypes between baseline and prior to stopping ART. In contrast, we observed three distinct time-dependent patterns of immune activation after ART was stopped (Fig. 4a). First, some immune cell activation occurred at the last off-ART pre-rebound timepoint (“PreR”). This included increases in the frequency of conventional dendritic cells [cDCs - non-cDC1/2 phenotype] expressing CD86 as well as CD56^hi^ NK cells expressing Perforin (Fig. 4b, top row). Second, multiple innate and adaptive immune cell types significantly upregulated activation markers (particularly CD71 on innate immune populations) just prior to and also early into rebound (“PostR1;” i.e., the first PBMC sample available after quantifiable rebound, sampled within 30 days of rebound and prior to viral loads exceeding 5,000 copies/mL [all were <2,600]; Fig. 4b, middle row). Third, multiple cell types exhibited immune activation only after rebound occurred. These changes included, for example, the expression of the antiviral transcription factor, T-bet, on non-naïve IgD-IgM+ B cells (Fig. 4b, bottom row).

For most of the above immune features profiled via CyTOF, viremic post-intervention controllers and non-controllers showed similar changes during peri-rebound period (i.e., PreR and PostR1; see blue versus red lines, respectively, in Fig. 4b; full dataset in **Supplementary Table 4**). However, although the overall abundance of plasmablasts did not consistently change in either group (Fig. 4c), the frequency of plasmablasts that express T-bet increased markedly at PostR1 only in the six viremic post-intervention controllers (median 1.18% at PreR versus 14.29% at PostR1) and not the non-controllers (4.00% versus 0.94%; Fig. 4d).

### Post-intervention control linked to in vivo proliferative CD8+ T cell response to rebounding virus

**Fig. 5.**
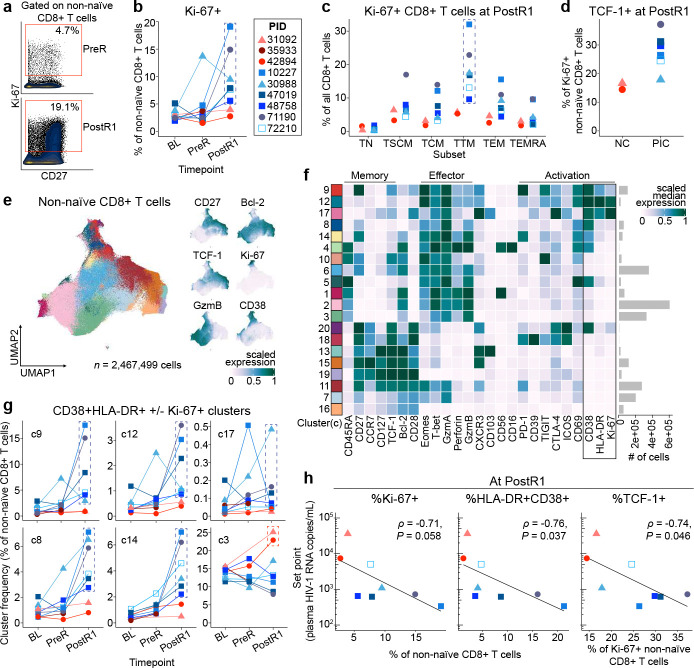
Post-intervention control of HIV is associated with a robust stem/memory-like, activated/proliferating CD8+ T cell response to rebound. a) Gating strategy and b) Frequency of Ki67+ non-naïve CD8+ T cells by manual gating at the BL, PreR, and PostR1 timepoints as measured by CyTOF. c) Distribution of Ki67+ cells across CD8+ T cell subsets at PostR1. d) Frequency of TCF-1+ cells within the Ki67+ non-naïve CD8+ T cells at PostR1. e) Left panel: UMAP of non-naïve CD8+ T cells including cells from all participants at BL, PreR, PreATI, and PostR1 timepoints. Right panels: Expression of individual markers on the UMAP. f) Heatmap of scaled median expression of markers across all clusters (activation markers CD38, HLA-DR, Ki-67 boxed for emphasis). g) Frequencies of clusters from (f) of activated CD8+ T cells across BL, PreR, and PostR1 timepoints. h) Spearman correlations between each participant’s post-ART set point viral load and the frequency of select non-naïve CD8+ T cell populations at the PostR1 timepoint. Analysis excludes PID 60610 (no rebound) and 35933 (no PostR1 sample). Dashed boxes in b, c, g encompass datapoints from post-intervention controllers. c, cluster. TCF-1, T cell factor 1. GzmB, Granzyme B. GzmA, Granzyme A. Eomes, Eomesodermin.

Strikingly, only post-intervention controllers had a robust increase in the frequency of activated non-naïve CD8+ T cells expressing Ki-67 (a cell cycle marker) early in response to rebounding virus (median [range] 2.65% [1.96–5.11] at Baseline versus 2.85% [1.83–13.71] at PreR versus 8.69% [5.78–19.11] at PostR1) compared to the non-controllers (2.60% [2.45–3.05] versus 2.63 [1.54–3.72] versus 3.33% [2.72–3.94]; Fig. 5a,b). A similar pattern was observed in the controllers using the combination of HLA-DR and CD38 as markers of activation (**Extended Data Fig. 5a**). The fraction of Ki-67+ CD8+ T cells at PostR1 in the controllers was highest in cells with a transitional memory phenotype (CD45RA−CD27+CCR7−; Fig. 5c), which is the phenotype of a majority of HIV-specific CD8+ T cells in PWH on ART.^35^

Three of the six post-intervention controllers also exhibited one or more of the following changes in their CD8+ T cells: an increase at the PostR1 timepoint in the frequency of total CD8+ T cells, total non-naïve CD8+ T cells, and/or HIV-specific CD8+ T cells as measured by ICS (**Extended Data Fig. 5b-e**). Additionally, some post-intervention controllers had an increase between baseline and pre-ATI in HIV-specific CD8+ T cell proliferation in response to *in vitro* stimulation with vaccine-matched (CE) or consensus sequence HIV clade B overlapping peptide pools (**Extended Data Fig. 5f-h**). However, some of these changes were also observed in the non-controllers. In contrast to the dynamic CD8+ T cell responses to rebound, there was no consistent increase in the frequency of HIV-specific CD4+ T cells by ICS or any type of CD4+ T cell expressing Ki-67 at PostR1 (**Extended Data Fig. 5i-l**).

### Control of HIV rebound associated with expansion of TCF-1+ stem/memory-like CD8+ T cells

We and others have found that individuals who control HIV spontaneously in the absence of ART (“elite” controllers) have HIV-specific CD8+ T cells that express high levels of the T cell memory-associated transcription factor, TCF-1, which supports their ability to robustly proliferate and differentiate into potent antiviral secondary effector cells in response to antigen stimulation.^35–39^ Here, TCF-1 expression was higher in the responding Ki-67+ non-naïve CD8+ T cells from post-intervention controllers compared to non-controllers at PostR1 (Fig. 5d). Unbiased clustering of the non-naïve CD8+ T cells further revealed that during rebound post-intervention controllers uniquely expanded multiple distinct, activated (i.e., co-expressing CD38 with Ki-67, HLA-DR, and/or CD69), CD45RA− non-naïve CD8+ T cell populations that express some effector proteins (e.g., Granzyme A, T-bet; see clusters [c]9, 12, 17, 8, 14; Fig. 5e, f, g and **Extended Data Fig. 6**). Some of these clusters more closely resemble a “precursor exhausted” (Tpex) phenotype (c9: TCF-1+, CD27+, PD-1+) while others have a more terminally differentiated effector phenotype (c14: Perforin+, Granzyme B+, c8: Perforin−, Granzyme B+). Notably, non-controllers did not expand any of these non-naïve CD8+ T cell populations. Instead, across timepoints, they tended to have a larger population of CD45RA+CCR7− (TEMRA phenotype) non-naïve CD8+ T cells that lack classical memory markers (c3; Fig. 5g).

Most notably, at PostR1, a higher frequency of activated non-naïve CD8+ T cells that express Ki-67 or HLA-DR/CD38 as well as a higher frequency of TCF-1+ cells within the Ki-67+ non-naïve CD8+ T cells was associated with the subsequent establishment of a low viral load set point post-ART (ρ = −0.71, *P* = 0.058; ρ = −0.76, *P* = 0.037; ρ = −0.74, *P* = 0.046, respectively; Fig. 5h). This is in contrast to the multiple virologic and immunologic factors measured prior to the ATI that did not correlate with viral load set point after ATI.

## Discussion

In this proof-of-concept mechanistic study of PWH who received a combination of immunotherapy interventions while on ART, we observed atypical HIV rebound kinetics and aviremic or low viremic control following ART interruption in seven of ten participants. Specifically, one post-intervention controller experienced no detectable rebound for more than 18 months off ART and six showed blunted rebound kinetics (similar to the kinetics observed in spontaneous HIV controllers taken off of ART) followed by maintenance of viral loads near or below 1,000 copies/mL for months. Although this study did not include a placebo arm, the proportion of participants who achieved this degree of sustained post-intervention control is substantially higher than what has been reported in recent HIV immunotherapy trials (~10–30% versus 70%).^8,9,11,12,14,15,17^ Notably, a recent study in NHPs has also demonstrated high rates of SHIV control after the administration of a similar combination therapy regimen relative to a placebo group.^6^ While there was no correlation between the establishment of post-intervention control and bNAb or lefitolimod exposure, HIV reservoir measurements, or immune responses up to the time of the treatment interruption, we observed a robust expansion of activated Ki-67+ CD8+ T cells in the peripheral blood early in response to rebounding virus uniquely in the viremic post-intervention controllers. There was a correlation between the frequency of the activated CD8+ T cells during rebound as well as their expression of the stem/memory marker TCF-1 and the establishment of a lower viral load set point. Rare control of HIV or SIV without ART or after stopping ART in the absence of other interventions has been associated with the presence of CD8+ T cells with strong proliferative responses to HIV/SIV antigen stimulation.^35–38,40,41^ To our knowledge, our study is the first to demonstrate that following a combination of immunotherapies, individuals who have a more robust *in vivo* CD8+ T cell response to emerging virus after stopping ART go on to establish better control of HIV.

In order to capture the early differences in CD8+ T cell responses to viral rebound, it was critical to evaluate a timepoint early after the start of rebound before viral loads diverged between the controllers and the non-controllers. Studies of acute-phase infections in mice and humans (including acute HIV) suggest that an overwhelming majority of the CD8+ T cells that we identified as becoming activated in response to rebound (i.e., those expressing Ki67 +/− HLA-DR +/− CD38) are likely HIV-specific.^42–44^ Additionally, they preferentially occupy a T cell memory subset typical for HIV-specific CD8+ T cells (transitional memory, TTM). Interestingly, at this early rebound timepoint, post-intervention controllers expanded populations of both activated TCF-1+ stem/memory CD8+ T cells as well as more terminally differentiated activated/cytotoxic (Granzyme B+) CD8+ T cells. Based on studies describing the dynamics of virus-specific CD8+ T cell subpopulations in chronic infections in mice,^45,46^ we hypothesize that the activated TCF-1+ CD8+ T cell population in the controllers serves as a longer-lived pool of stem-like CD8+ T cells that sustains the response against chronic viremia by continually giving rise to short-lived cytotoxic effector cells that kill HIV-infected cells. These observations closely mirror human studies in other settings that have found a correlation between *in vivo* CD8+ T cell proliferative responses in the blood and/or the presence of a larger responding TCF-1+ CD8+ T cell population and positive clinical outcomes (e.g., SARS-CoV-2 clearance^47,48^ or cancer response to immunotherapy^49,50^ In this light, the results of this study support continued efforts towards optimizing therapeutics to promote effective T cell control of HIV.

These findings raise an important question: how did the therapeutic interventions enable a highly effective *in vivo* CD8+ T cell response against HIV rebound? The immunologic changes during each of the three stages of the study suggest that a combination of therapeutic vaccination and bNAbs administered at the time of treatment interruption may have been particularly impactful, although we cannot rule out an effect of the administration of the bNAbs plus the TLR9 agonist. The DNA/MVA therapeutic vaccine regimen in Stage 1 of the study boosted or elicited new HIV-specific CD8+ T cells targeting conserved elements in Gag in nine out of ten participants, and the administration of the two bNAbs at the time of treatment interruption in Stage 3 was associated with broad innate and adaptive immune activation prior to rebound. In contrast, we could not detect consistent evidence of latency reversal or immune activation during Stage 2 when the TLR9 agonist was administered together with the bNAbs during ART suppression. Our results are consistent with emerging data from other studies in which infused bNAbs (given either at the time of ART initiation or interruption) can enhance the quality of HIV-specific CD8+ T cell responses.^18^ This may occur either via a classical “vaccinal effect” mediated by bNAb:HIV immune complexes, and/or because the slow washout of bNAbs given concurrently with ART interruption blunts the typically explosive rebound viremia, thus enabling CD8+ T cells to out-compete the virus by being more optimally stimulated (boosted +/− newly primed) under low-antigen conditions.^8^ While our small proof-of-concept study argues that a combination approach might prove more effective than one including bNAbs alone, this hypothesis needs to be tested in larger, placebo-controlled, randomized controlled trials.

We did not identify specific immune or viral features prior to rebound that predicted post-ART outcomes. Specifically, the magnitude and *in vitro* proliferative capacity of HIV-specific CD8+ T cell responses did not correlate with post-ART viral load set points, suggesting that these *in vitro* measures of CD8+ T cell functional capacity do not accurately reflect their *in vivo* response potential. Furthermore, both post-intervention controllers and non-controllers had similar patterns of broad innate and adaptive immune activation around the time of rebound. Measuring other immune or viral parameters peri-rebound (e.g., autologous antibody activity, which is not possible with current assays in the presence of bNAbs) may be required to distinguish between these groups. Additionally, deeper characterization of CD8+ T cell responses (e.g., by epitope mapping, measuring T cell responses against autologous virus sequences, and tracking T cell clonotype dynamics) together with paired analysis of innate immune activation, particularly at more timepoints during the period of bNAb-mediated viral suppression leading up to rebound as well as in tissue sites of reservoir persistence, will be critical to further explore potential mechanisms and possibly identify biomarker(s) that can be used to predict responders to combination immunotherapy before rebound.

Although seven of our study participants had initiated ART early after HIV acquisition, the proportion of post-intervention controllers exceeds what has been observed after stopping ART in other early-treated observational cohorts (10–20%).^51,52^ The aviremic controller (PID 60610) had amongst the lowest measures of cell-associated HIV DNA or RNA. Prior to participation in this trial, this individual was described in a study of two people who were identified and treated with ART very early after acquiring HIV (“Participant B”).^33^ In these cases, early ART initiation may have prevented reservoir establishment but also restricted long-term HIV-1 immune responses; the other early-treated participant in that study rebounded approximate 8 months after pausing ART. Very early initiation of ART, as in the RV144 cohort^40^ and in our own experience,^33^ may result in delays in viral rebound, but never as long as 18 months, suggesting that both a low reservoir plus sustained treatment-induced host responses may be required to maintain aviremia.^33,40,53–55^ Mathematical modeling supports this concept.^56^

This study has important limitations. First, the trial was small and uncontrolled. We made a deliberate decision to use a single-arm, proof-of-concept approach because of the complexity of the combination intervention, the impracticality of maintaining blinding for participants and investigators, the goal of assessing safety in humans, ethical concerns regarding the use of a placebo group, and the established body of evidence of historical rates of post-treatment and post-intervention control in other studies. We aimed to demonstrate proof-of-concept through detection of a large effect in comparison to the established literature. As a result, our findings require replication in larger cohorts, some of which are already ongoing or planned (NCTs 04319367, 05245292, 04340596, 06484335, 04983030, 06071767, 06205602, 05719441, 05300035). Second, study pauses due to the COVID-19 pandemic as well as the FDA-mandated safety pause resulted in variability in how the second stage was conducted. Still, all participants received each of the interventions and viral rebound kinetics did not appear to differ based on differences in the clinical schedules. Finally, the ability to detect changes in HIV DNA and RNA was limited by the low detection frequencies and levels at baseline.

In summary, we observed a higher-than-expected proportion of participants achieving durable control of HIV, with low post-ART viral load set points (or undetectable plasma virus) maintained over many months off ART. This finding provides proof-of-concept for the design of future combination immunotherapy interventions in people with the goal of inducing a potent and effective immune response that can achieve sustained ART-free HIV remission.

## Methods

### Participants

Combination immunotherapy trial (UCSF-amfAR study; NCT04357821): Participants were recruited from the University of California, San Francisco-based SCOPE cohort (NCT00187512), which enrolls and prospectively measures adults living with HIV. Briefly, interested SCOPE participants completed a screening visit at which their eligibility for the trial was assessed. The trial enrolled adults (age 18–65 years) with a history of confirmed HIV infection on antiretroviral therapy (ART) for at least 12 months without any interruptions of more than 14 consecutive days within the preceding year, who were on a stable regimen that did not include a non-nucleoside reverse transcriptase inhibitor (NNRTI) for at least the four weeks prior to enrollment. Those who were known to have spontaneously controlled HIV prior to ART initiation were not eligible. Participants were required to have plasma HIV RNA levels below the limit of quantification on all available determinations in the preceding 24 months (“blips,” or isolated single values above the level of quantification but <200 copies/mL were allowed), with screening CD4+ T cell count ≥500 cells/μL. We excluded participants who had a CD4+ T cell nadir <350 cells/μL, active hepatitis B or C infection, or other comorbidities (see Supplemental Methods for additional screening and exclusion criteria). Although participants with clinical evidence of pre-ART spontaneous control were ineligible, one individual had an HLA allele associated with spontaneous control of HIV (HLA-B*57:01^57^). Importantly, we screened for susceptibility to the two broadly neutralizing antibodies and excluded those determined to have significantly reduced phenotypic susceptibility to 10–1074 and VRC07–523LS using the PhenoSense Monoclonal Antibody (mAb) Assay (Labcorp-Monogram Biosciences), defined as IC50 values in the top 10% for at least one of the antibodies (10–1074, >50 μg/mL; VRC07–523LS, >0.3834 μg/mL) or in the top 25% for both antibodies (10–1074, >0.1386 μg/mL; VRC07–523LS, >0.2024 μg/mL), based on values derived from a prior assessment in another, unrelated study (A5257).^58^

Prospective ATI study (SCOPE-ATI study; NCT04359186): see Supplemental Methods.

Both studies were approved by the UCSF IRB. All participants were counseled extensively regarding the risks of HIV transmission during the treatment interruption^59^ and could opt into socio-behavioral research.^60,61^ Because the studies occurred in the context of the COVID-19 pandemic, extensive plans to address COVID-19 risk mitigation^62^ and accommodate SARS-CoV-2 vaccination^63,64^ were developed.

### Immunotherapy trial design

The study interventions are shown in Fig. 1. At baseline and approximately 4 weeks later, participants received a DNA vaccine, which included a p24 conserved element (CE) 1/2 plasmid (4 mg) with an IL-12 DNA plasmid (2 mg) split into two separate injections administered via electroporation using the TriGrid Delivery System (TDS-IM v1.0; Ichor Medical Systems). At 12 weeks, they received the p24CE 1/2 plasmid (4 mg) with a p55gag plasmid (2 mg) and IL-12 plasmid (2 mg) split into two separate injections administered via electroporation. At approximately week 20, participants received an MVA62B boost (10^8^ tissue culture infectious dose [TCID]_50_/mL 1mL) via intramuscular injection. At week 24, participants received intravenous infusions of 10–1074 (30 mg/kg) and VRC07–523LS (20 mg/kg). From weeks 25–33, participants received weekly subcutaneous injections of 60 mg of lefitolimod (2 injections per dose, 1–9 doses, see “Safety”). At approximately week 34, they received a second infusion of each of the two bNAbs and 48 hours later interrupted ART. Clinical laboratory values, including plasma HIV RNA levels, were checked weekly from week 34–58 and then at least every other week for the remainder of the ATI. ART was reinitiated for any one of the following outcomes: (1) plasma HIV RNA >50,000 copies RNA/mL for four weeks, (2) plasma HIV RNA >10,000 copies RNA/mL for 6 weeks, (3) plasma HIV RNA > 2000 copies RNA/mL for 12 weeks, or (4) plasma HIV RNA > 400 copies RNA/mL for 24 weeks. ART could also be reinitiated for confirmed CD4+ T cell decline below 350 cells/μL, the presence of severe acute retroviral syndrome, concerns about HIV transmission risk, acute SARS-CoV-2 infection or need for COVID-19 or Mpox vaccination, or at the request of the participant or their primary care clinician. Following reinitiation of ART, participants were seen at approximately 1, 2, and 6 months to confirm virologic suppression.

### Clinical measurements

At each visit, participants underwent an assessment for adverse events, which were categorized in terms of relatedness (definitely, probably, possibly, or not related) and severity using the NIH Division of AIDS Table for Grading the Severity of Adult and Pediatric Adverse Events, Corrected Version 2.1 (July 2017). Blood was collected for safety testing as outlined in Supplemental Methods. Plasma HIV RNA levels were initially measured using the Abbott Real Time HIV-1 PCR (limit of quantitation 40 copies/mL) and subsequently with the Hologic Aptima assay (limit of quantitation 30 copies/mL); for the purposes of this analysis, all plasma HIV RNA levels <30 copies/mL were considered unquantifiable. All laboratory measurements were performed in the clinical laboratory at Zuckerberg San Francisco General Hospital. The beginning of viral rebound (day 0) was defined as the first day of two consecutive quantifiable plasma viral loads. Peak viral load following rebound was defined as the maximum viral load within the first six weeks after time of rebound. The slope of viral load rebound was defined as the slope of a linear regression between time of rebound and time of peak viral load for each participant. Post-rebound viral set points were defined as the median of the viral loads measured starting two weeks after the peak viral load until ART restart. See **Supplementary Table 2** for participant-level viral load outcomes and **Supplementary Table 3** for complete post-ATI viral load dataset.

### Biospecimen collection and storage

At most study visits, peripheral blood was collected by standard blood draw or leukapheresis and stored as serum, plasma, and viable cryopreserved peripheral blood mononuclear cells (PBMCs), as described previously.^35^ Serum and plasma were stored at −80°C and PBMCs were stored in liquid nitrogen.

### Plasma levels of antiretroviral drugs during ART interruption

For 2–3 timepoints per participant throughout the study-defined ART interruption, plasma antiretroviral levels were measured using NIH Clinical Pharmacology Quality Assurance (CPQA)-approved high performance liquid chromatography tandem mass spectrometry (LC-MS/MS) or ultra high performance liquid chromatography with a photodiode array detector (UPLC-PDA) methods, previously described (tenofovir^65^) or detailed in Supplemental Methods (emtricitabine and dolutegravir assays).

### 10–1074 and VRC07–523LS levels and susceptibility testing

The 10–1074 and VRC07–523LS PK assays were performed at the NIH Vaccine Research Center, using a previously described Meso Scale Discovery (MSD)-based platform and as described in Supplemental Methods.^66^ The PhenoSense mAb Assay (Labcorp-Monogram Biosciences, South San Francisco, CA, USA) was used to evaluate susceptibility of autologous HIV to neutralization by 10–1074 and VRC07–523LS. Full-length envelope sequences were amplified from either cell-associated HIV DNA at baseline or pre-rebound timepoints or plasma-derived HIV RNA post-rebound and cloned into an envelope expression vector. Pseudovirion producer cells were co-transfected with envelope expression vectors and a HIV genomic reporter vector, with *env* replaced by luciferase. Luciferase reporter pseudovirions were harvested and tested for susceptibility to 10–1074 and VRC07–523LS in a cell-based neutralization assay. The concentration of antibody required to inhibit infection by 50% or 90% (IC_50_, IC_90_) as well as the maximum percent inhibition (MPI) was assessed (see **Supplementary Table 3** for full dataset).

### HIV DNA and RNA measurements

CD4+ T cells were isolated from cryopreserved PBMCs (5×10^6^-3×10^7^ cells) by immunogenic negative selection (StemCell Technologies, Vancouver, Canada) and then counted. Total cellular DNA and RNA were extracted from each sample using TRI Reagent^®^ (Molecular Research Center, Inc, Sunnyvale, California, USA). The extracted DNA samples were fragmented using QIAshredder columns (Qiagen, Redwood City, CA, USA).^67^ DNA and RNA concentrations were measured using a NanoDrop One microvolume spectrometer (Thermo Fisher Scientific, Waltham, MA, USA). The Intact Proviral DNA Assay (IPDA) was performed in a duplex ddPCR assay (FAM and VIC), as described previously (450–750 ng of DNA input per well, 7–8 replicates per sample),^32,68^ and HIV transcription profiling was performed using a modification of previously described methods.^69^ Both assays are described fully in Supplemental Methods.

### Intracellular cytokine staining

The magnitude and phenotype of HIV-specific CD4+ and CD8+ T cells was characterized by flow cytometry using intracellular cytokine staining (ICS). Cryopreserved PBMCs were thawed and rested overnight at 37°C 5% CO_2_. One million cells per condition were incubated for 6 hours at 37°C and 5% CO_2_ with HIV peptide pools at a concentration of 1 μg/mL per peptide (full or individual smaller CE peptide pools [provided by BKF], or HIV clade B Gag, Pol, Nef, Env overlapping peptide pools [from BEI Resources NIH/NIAID, Manassas, VA, USA]), SEB (positive control), or R10 media plus DMSO as a negative control in the presence of brefeldin A (Sigma-Aldrich, St. Louis, MO, USA), monensin (Sigma-Aldrich), and anti-CD107a antibody (BD Biosciences, Milpitas, CA, USA). Cells were then harvested and first stained in PBS with viability stain (Zombie UV; Biolegend, San Diego, CA, USA) for 10 minutes at 25°C followed by surface staining markers in PBS+1%FBS with Brilliant Stain Buffer Plus (BD Biosciences) for 30 minutes at 25°C. Following surface staining, cells were fixed and permeabilized for 45 minutes at 4°C and then stained with intracellular antibodies overnight at 4°C using the eBioscience Foxp3/Transcription Factor Staining Buffer Set according to the manufacturer’s protocol (Thermo Fisher Scientific). See **Supplementary Table 6** for flow cytometry panels. Samples were resuspended in PBS+1%PFA and stored at 4°C before acquiring on a Cytek 5L Aurora (Cytek Biosciences, Fremont, CA, USA). Compbead anti-mouse, -rat and -hamster particles (BD Biosciences) were stained as reference controls for unmixing except that heat-killed PBMCs were used as a Zombie UV staining unmixing reference. Longitudinal samples from the same participants were run on the same experiment day. To measure inter-experiment variability, PBMCs from the same donor without HIV were stimulated with SEB (or incubated with DMSO) and stained in parallel for each experiment. HIV-specific T cell responses were measured based on the fraction of total CD4+ or CD8+ T cells that expressed IFNɣ after peptide stimulation and after subtraction of background IFNɣ staining present in a control unstimulated well containing only an equivalent concentration of DSMO. “Total” HIV-specific T cell responses were calculated based on the summed IFNɣ+ cells after stimulation with Gag, Pol, Nef, or Env overlapping peptide pools.

### *In vitro* HIV-specific T cell proliferation

Thawed PBMCs in R10 media were rested overnight at 37°C and 5% CO_2_. Cells were then labeled with 0.5 mM CTV (Thermo Fisher Scientific) according to the manufacturer’s instructions. One million CTV-labeled cells were stimulated for 6 days at 37°C with 5% CO_2_ in R10 media plus DMSO (negative control) or R10 containing 0.1 μg/mL HIV peptide pools composed of peptides derived from Gag conserved element sequences, HIV clade B overlapping peptide pools (as described above; from BEI Resources) or broad T cell activation as a positive control (ImmunoCult human CD3/CD28 T cell activator; StemCell Technologies) per manufacturer’s instructions. After stimulation, the cells were stained for surface and then intracellular proteins and then analyzed as described above. See **Supplementary Table 6** for flow cytometry panels. Proliferative responses were measured as the fraction of CD4+ or CD8+ T cells that diluted CTV (i.e., %CTV^lo^) after stimulation with HIV peptide pools. Background proliferation present in the negative control wells was subtracted.

### Mass cytometry (CyTOF)

CyTOF analyses were performed over the course of five separate experiments, as described previously.^34^ PMBCs were thawed and only samples with >70% viability were used for analysis. We stained 2–4 million cells per panel in two mass cytometry panels (see **Supplementary Table 6** for CyTOF panels), following a previously published protocol^70^ with modifications noted in Supplemental Methods.

### Bulk RNA sequencing

Bulk RNA sequencing was performed on PBMCs that had been cryopreserved and then thawed. RNA was isolated using the RNeasy Micro Kit (Qiagen) following the manufacturer’s instructions. Total RNA was quantified using Qubit RNA Assay Kit and RNA quality was assessed using Agilent 4200 TapeStation instrumentation (Agilent, Santa Clara, CA, USA). mRNAseq libraries were constructed using KAPA mRNA HyperPrep Kit with an input of 50 ng total RNA. Qubit DNA assay kit was used to determine library concentration and Agilent 4200 TapeStation instrumentation was used to determine library size distribution and quality. Equimolar amounts of sample were pooled prior to loading onto an Illumina NovaSeq 6000 instrument (Illumina, Foster City, CA, USA) for sequencing. Samples were demultiplexed using bcl2fastq (Illumina). Adapters with low-quality ends were trimmed from FASTQ files with Trim Galore (v0.6) and quality analysis performed by FastQC (v0.11.2). Reads mapping to ribosomal RNA and globin were removed using Bowtie2 (v2.4.2),^71^ resulting in 25 million reads per sample for further analyses. Remaining sequences were aligned to the human GRCh38 genome (Ensembl v112) using STAR (v2.7.10b).^72^ Alignment results showed >90% of reads mapped to the human genome.

### Statistical analyses

#### Slope of HIV rebound modeling.

Linear mixed effects modeling was performed in R (v4.3) using the lmerTest package (v3.1; two-sided t-test with Satterthwait’s approximation for degrees of freedom) for viral rebound curves from the time of rebound to the time of peak viral load (as defined above: the highest viral load within the first six weeks post-rebound) or the time of ART restart if that came first, with fixed effects for post-rebound group on slope of rebound and random effects for between-participant variability.

#### PK and PK-PD analyses.

We described plasma bNAb PK using population PK modeling approaches (including data from all ten participants) in Monolix software (Lixoft v2023R1) using 1- and 2-compartment models for 10–1074 and VRC07–523LS, respectively, including participant-specific variation in clearance (both bNAbs) and volume of the central compartment (VRC07–523LS only). Residual variability was described using a proportional error model. Model details are included in **Supplementary Table 7.** We performed two-tailed Spearman’s correlations to determine the relationship between bNAb exposure (area under the concentration-time curve, AUC; peak concentration, C_max_), bNAb susceptibility (post rebound IC_90_), and rebound kinetics (time to rebound, post-ART set point for the nine participants who experienced viral rebound).

#### ICS analysis.

Changes in frequencies over time were determined using linear mixed effect analysis with two-tailed Tukey’s multiple comparison’s test (GraphPad Prism, Boston, MA, USA). See **Supplementary Table 4** for full background-subtracted dataset.

#### CyTOF manual gating analysis.

Following data acquisition, normalization and de-barcoding of samples was performed using Premessa (https://github.com/ParkerICI/premessa). FCS files were imported into CellEngine (CellCarta, Montreal, Canada). Traditional hierarchical gating was applied to identify 12 standard ^34^ “landmark” immune populations and 27 “sub-landmark” populations (see **Extended Data Fig. 4a** for gating strategy and **Extended Data Fig. 4b,c** for the identity and longitudinal frequency of these populations). Within each of the “parent” cell types, we manually gated positive and negative populations of biologically relevant phenotypic markers from the two mass cytometry panels (see **Supplementary Table 4** for full dataset). For each of the parent cell types, we only included phenotypic markers for which we could clearly gate a positive population above background antibody staining levels. Batch effects were minimized by using PBMC control samples in each experiment from people without HIV. For the final analysis, we excluded phenotypic features present at very low frequencies (i.e., we only included phenotypic features for which at least 10% of the samples had >5% marker+; a total of 303 features were included in the testing). To determine significant changes in the manually gated landmark, sub-landmark, and phenotypic features over time, the frequency of each feature as a percent of the parent population for each sample was exported and log10 transformed with a constant of 1×10^6^. Two-tailed Wilcoxon signed rank tests were performed on the log-transformed values for timepoint comparisons (PreATI, PreR, or PostR1 each compared to Baseline) for the six viremic post-intervention controllers only. Due to the small sample size, we did not adjust for covariates and the significant *P* values for the changes in features over time were not adjusted using multiple testing correction methods.

#### CyTOF clustering analysis of non-naïve CD8+ T cells.

For clustering analysis of non-naïve CD8+ T cells, all manually gated live, intact, CD45+ single cells (see gating strategy) were downloaded as FCS files from CellEngine and first batch corrected using cyCombine (https://github.com/biosurf/cyCombine). Batch corrected files were reuploaded to CellEngine and gated on total CD8+ T cells (see gating strategy, **Extended Data Fig. 4a**). The FCS files containing non-naïve CD8+ T cells were used in downstream clustering analysis using Catalyst (https://github.com/HelenaLC/CATALYST) which performs clustering utilizing FlowSOM and ConsensusClusterPlus. Non-naïve CD8+ T cell clusters were selected for downstream analysis. For detailed clustering and visualization settings, see Supplemental Methods.

#### Bulk RNAseq Differential Gene Expression analysis.

Statistical processing and analysis of RNAseq count data was performed in the R statistical computing environment (R core team 2023). Genes were filtered by a cross sample mean of 50 or greater and then normalized using edgeR with calcNormFactors using trimmed mean of M-values (TMM^73^) normalization and log2 normalized with voom.^74^ Differential Expression (DE) analysis was performed on baseline samples between viremic controllers (*n* = 6) and non-controllers (*n* = 3) using lmfit through limma.^75^ Significance cut-offs were set at log2 fold change >1.5 and nominal *P* value <0.05.

## Supplementary Material

Supplement 1

## Data Availability

Bulk RNAseq data available here: GSE288962; reviewer token ufwvoigylpmjfkn Full processed (gated) CyTOF and flow cytometry data available in **Supplementary Table 4.** Raw CyTOF and flow cytometry data available upon request and full data will be made available upon publication.
