# Supplementary material for: Combination immunotherapy induces post-intervention control of HIV
Source: Res Sq. 2025 Mar 19:rs.3.rs-6141479. Preprint. [Version 1] doi: 10.21203/rs.3.rs-6141479/v1 (PMC11957202; doi:10.21203/rs.3.rs-6141479/v1)
Supplement: Supplement 1 [file NIHPPrs6141479v1-supplement-1.pdf]

Supplemental methods

Trial participants

Immunotherapy trial - Additional screening criteria: creatinine clearance >60 mL/min via the Cockroft-Gault method, absolute neutrophil count ≥ 1000 cells/μL, hemoglobin ≥ 10 g/dL, platelets ≥ 100,000/μL, aspartate aminotransferase (AST) and alanine aminotransferase (ALT) <= 2 times the upper limit of normal, and total and direct bilirubin less than or equal to the upper limit of normal. *Additional exclusion criteria:* history of chronic liver, kidney, cardiovascular, clotting/bleeding, neurologic, ocular, allergic or immune-mediated medical conditions, or serious psychiatric comorbidities, those with any history of HIV-associated malignancy or those with non-HIV associated malignancy in the last 36 months, those with recent

hospitalization, those for whom administration of the vaccines via electroporation would be technically difficult, and those who were pregnant, breastfeeding, or unwilling to use contraception during the study.

Observational ATI study: Concurrent with the combination immunotherapy trial, participants from SCOPE also had the opportunity to be screened for an intensively monitored analytic treatment interruption study that did not include any pharmacologic intervention (SCOPE-ATI; NCT04359186). Participants in this parallel study were adult PWH (age >18 years) on stable ART, with plasma HIV RNA levels below the limit of quantification on all available determinations in the preceding 12 months and screening CD4+ T cell counts ≥ 350 cells/µL. We excluded participants with comorbidities that would preclude a brief ATI and those who were pregnant, breastfeeding, or unwilling to use contraception during the study. Prior spontaneous controllers were defined as individuals who upon investigator assessment had been able to achieve complete or partial control of HIV with set points generally <2,000 copies/mL in the absence of ART, and who had initiated ART in the setting of loss of control or evolving clinical practice guidelines supporting treatment of all PWH. Following a series of baseline measurements, participants interrupted ART and were monitored three times weekly until the plasma HIV RNA level was confirmed to be above 200 copies/mL. Those who were not known to be prior controllers then resumed ART while prior controllers were given the option of extending the ATI to achieve set point, with similar virologic restart criteria as the combination immunotherapy trial.

Study products

The p24CE1/2 pDNA vaccine[23,26,27,76] was designed to target immune responses to conserved elements (CE) of the HIV-1 Gag protein based on stringent conservation, broad HLA-coverage and association with HIV control.[77–81] The drug product was provided as single use vials containing sterile 4 mg/ml buffered solution. The $p55^{gag}$ pDNA (plasmid code 114H) spans 5518 bp and encodes a full-length $p55^{Gag}$ protein from the HIV-1 molecular clone HXB2 (clade B; GenBank NP_057850.1). The drug product was provided as single use vials containing sterile 4 mg/ml buffered solution. The GENEVAX® *IL-12* pDNA is a dual promoter expression plasmid that expresses the two genes encoding human IL-12 subunits p35 and p40 under separate regulatory control.[82–87] The drug product was provided as single use vials containing sterile 2 mg/ml buffered solution. MVA62B is a highly attenuated double recombinant MVA vaccine consisting of an MVA vector and two vaccine inserts (HIV-1 clade B Gag and Pol proteins, HIV-1 clade B (ADA) Env).[88–90] The drug product was provided as single use vials containing buffered solution in a concentration of 10^8 $TCID_{50}$/mL. Lefitolimod is a synthetic DNA-based immunomodulatory molecule with two single-stranded loops separated by a double-stranded stem.[91,92] The drug product was provided as single use vials containing sterile (15 mg/mL) buffered solution. 10-1074 is a recombinant, fully human monoclonal antibody (mAb) of the IgG1λ isotype that specifically binds HIV-1 glycoprotein protein 120 (gp120;

specifically, it recognizes the base of the V3 loop and surrounding glycans on the HIV-1 Envelope).[28,93,94] The drug was supplied as single use vials containing sterile 20 mg/ml buffered solution. VRC07-523LS is a highly potent and broadly neutralizing monoclonal antibody directed against the CD4-binding site of gp120.[95,96] The LS designation specifies methionine to leucine (L) and asparagine to serine (S) (M428L/N434S, referred to as LS) changes within the C-terminus of the heavy chain constant region. The LS mutation was introduced by site-directed mutagenesis to increase the binding affinity for the neonatal Fc-receptor (FcRn), resulting in increased recirculation of functional IgG,[97,98] thus increasing plasma half-life. The drug product was provided as single use vials containing sterile 100mg/mL buffered solution.

Safety testing

Blood tests for safety included metabolites, renal and hepatic function tests, complete blood counts and differentials, and CD4+ T cell counts.

ddPCR to assess intact and defective proviruses

Detailed methods: DNA from each sample was tested in seven to eight replicate ddPCR wells with a DNA input of 450-750 ng per well. HIV DNA levels were normalized to copies/million CD4+ T cells by measuring the human gene copy numbers in a separate duplex ddPCR assay for two regions of the human RPP30 gene.[68] Intact HIV DNA levels were further corrected for DNA shearing and/or drop-out by using the data from the RPP30 assay, as calculated using the equation:

$$corrected\ intact\ HIV\ DNA = measured\ intact\ DNA \times \frac{1}{1-\left(\frac{Avg(Q1+Q4)}{Q2+Avg(Q1+Q4)}\right)}$$

where Q2 is the number of droplets in the FAM+VIC+ quadrant, Q1 is the number of droplets in the FAM+VIC- quadrant, and Q4 is the number of droplets in the FAM-VIC+ quadrant from the RPP30 assay.[68] DNA samples from blood donors without HIV were used as negative controls for the IPDA and as positive controls for the RPP30 assays. For additional rigor, HIV DNA levels were also normalized to copies per million CD4+ T cells using the total DNA input per well, assuming that 1μg of DNA is equivalent to 160,000 cells,[99] with similar results (data not shown).

HIV transcription profiling

Detailed methods: 200-500 ng of each RNA sample was used to measure total initiated HIV transcripts using an initial polyadenylation reaction (necessary for reverse transcription of short HIV transcripts containing only the Trans Activation Region [TAR]), followed by reverse transcription (RT) and duplicate ddPCR assays for the TAR region.[69] For the detection of HIV RNA regions other than TAR, 1200-5000 ng of RNA were reverse transcribed without polyadenylation in a common RT reaction using random hexamers and poly-dT.[69] Levels of the other HIV RNA regions were measured using duplicate aliquots (5 μL) from

this common RT reaction. Primers/probes and ddPCR conditions were as described previously,[69] except that we performed 3 different duplex ddPCR reactions to measure: 1) 5’ elongated (R-U5-pre-Gag or “Long LTR,” in FAM) and 3’ or distal-transcribed (Nef, in VIC) HIV RNA; 2) mid-transcribed, unspliced (Pol, in FAM) and multiply spliced TatRev (in VIC) HIV RNA; and 3) and completed (U3-polyA, or “PolyA”, in FAM) and multiply spliced TatRev (in VIC) HIV RNA. All ddPCR plates included two positive controls (standards prepared from supernatant “virion” RNA and an in vitro transcribed, multiply spliced, polyadenylated HIV RNA)[69] and RNA from uninfected blood donors as the negative control. HIV RNA levels were normalized to 1 μg of total cellular RNA, the equivalent of approximately a million cells, using the RNA concentration and inputs into the RT and ddPCR wells.

Mass Cytometry (CyTOF) *Detailed methods:* We marked dead cells by incubating the samples for one minute with 25 mM cisplatin (Sigma-Aldrich) in phosphate buffered saline (PBS) plus EDTA, performed surface staining with metal-tagged antibodies in PBS with 0.5% bovine serum albumin (BSA) for 30 min at room temperature, fixed and permeabilized cells following manufacturer’s instructions for the eBioscience Foxp3/Transcription Factor Staining Buffer Set (Thermo Fisher Scientific), barcoded samples using mass-tag cellular barcoding reagents diluted in Maxpar Barcode Perm Buffer (Standard BioTools, South San Francisco, CA, USA) as described previously,[70] combined up to twenty barcoded samples into a single tube, performed intracellular staining with antibodies diluted in eBioscience Foxp3/Transcription Factor kit perm wash (Thermo Fisher Scientific), fixed cells in freshly prepared 2% paraformaldehyde (Electron Microscopy Sciences, Hatfield, PA, USA) in the presence of a DNA intercalator,[100] and then washed and ran cells on the Standard BioTools CyTOF 2 mass Cytometer within one week of staining.

Detailed clustering methods: CD8+ T cell clusters were generated using the following markers: Bcl-2, CCR7, CD103, CD127, CD16, CD25, CD27, CD28, CD38, CD39, CD45RA, CD56, CD69, CD95, CTLA-4, CXCR3, CXCR5, Eomes, Granzyme A, Granzyme B, HLA-DR, ICOS, PD-1, Perforin, T-bet, TCF-1, and TIGIT. 20 cluster populations were obtained using FlowSOM (10x10 grid) to generate 100 SOM codes followed by meta clustering using ConsensusClusterPlus. One cluster containing naïve CD8+ T cells based on the positive expression of CD45RA, CD27, and CCR7 was removed and cells were re-clustered to obtain 20 non-naïve CD8+ T cell clusters. UMAP was used for dimensionality reduction and ran using 10,000 cells, including the markers used in clustering plus Ki-67. All plots displaying CyTOF data were generated using Catalyst, ggplot2 (https://github.com/tidyverse/ggplot2), or ComplexHeatMap (https://github.com/jokergoo/ComplexHeatmap).

Antiretroviral measurements

Detailed methods: Tenofovir (TFV) was fortified with a deuterated internal standard, and then 50 μL of

sample was extracted by protein precipitation with acetonitrile. The sample extracts were separated on a Phenomenex Synergi Polar-RP high performance liquid chromatography (HPLC) column (150 × 2.0mm, 4µm; Phenomenex, Torrence, CA, USA), then detected on a Sciex API 5000 mass spectrometer (Sciex, Redwood City, CA, USA). The lower limit of quantification (LLOQ) for TFV was 5 ng/mL, with a calibration range of 5–1000 ng/mL. An emtricitabine (FTC) assay was developed on the API 5000 with a calibration range from 5-2500 ng/mL and plasma samples were prepared with simple protein precipitation with acetonitrile before injection onto a Phenomenex Synergi Polar-RP HPLC column (2.0x150mm 4µm) for LC-MS/MS analysis. The dolutegravir (DTG) assay was developed on a Ultraperformance Liquid Chromatography-Photodiode Array (UPLC-PDA) system (Waters, Milford, MA, USA) with a calibration range of 100 to 10000 ng/mL, and samples were prepared with solid-phase extraction using a hydrophilic-lipophilic balance (HLB) microelution plate before being injected onto a bridged ethyl hybrid (BEH) C18 LC column (50x2.1 mm, 1.7 µm).

VRC07-523LS and 10-1074 levels

Briefly, bNAb respective anti-idiotypic (ID) antibody solutions were coated on Meso Scale Discovery (MSD) 96-well bare plates. Plates were sealed and placed at 4ºC overnight. The next day, plates were washed then blocked. The blocking solution was washed and reference and/or test samples were applied to the wells and allowed to incubate with shaking. Plates were washed to remove unbound sample. Sulfo-tag labeled anti-human IgG detection antibody was applied to the wells and allowed to associate with complexed anti-ID and bNAb within the assay wells. Plates were washed to remove unbound detection antibody. A read solution containing electrochemiluminescence (ECL) substrate was applied to the wells, and the plates were entered into the MSD Sector instrument. A current was applied to the plates and areas of well surface which form a full anti-ID/bNAb/anti-human IgG-SulfoTag complex emitted light in the presence of the ECL substrate. The MSD Sector instrument quantitates the amount of light emitted and reports this ECL unit response as a result for each sample and standard of the plate. The amount of bNAb sandwiched by the anti-ID and anti-human IgG antibodies is directly proportional to the concentration of reactive bNAb in the sample wells. The sample signal is interpolated and dilution-adjusted relative to the reference standard. All calculations are performed within Excel and GraphPad software. The limits of detection are 0.156 µg/mL for VRC07-523LS and 0.312µg/mL for 10-1074.

Anti-drug antibody levels

We performed competitive and functional ADA assays for both bNAbs. A three-level tiered approach was used to screen, confirm, and functionally characterize the anti-drug antibody (ADA) in the clinical samples for VRC07-523LS and 10-1074. Each bNAb was assayed separately. The first tier of testing was a screening assay performed on the MSD platform. Sample and plate controls were diluted 1:2 and incubated with

optimized concentration of SulfoTag-labelled bNAb (reporter molecule) and biotinylated bNAb (capture molecule). The incubated sample-mixture was then added to a pre-blocked streptavidin coated MSD plate. Any ADA present in the sample bound both the biotinylated and SulfoTag labeled forming a bridging complex attaching to the streptavidin-coated MSD plate. ECL was measured by an MSD plate reader. A test sample was tier 1 positive if the ECL is greater than the floating positivity cut point. If a sample tested positive in tier 1, tier 2 testing was performed on the sample. The second tier of testing was a competitive assay performed on the MSD platform. In the tier 2 assay, the sample was pre-incubated with and without the unlabeled therapeutic mAb and evaluated for the reduction (percentage) of signal in the presence of unlabeled bNAb at 10 µg/mL. Sample was incubated for 60 minutes at 37°C. Subsequently, this mixture was incubated with SulfoTag-labeled bNAb (reporter molecule) and biotinylated bNAb (capture molecule). Subsequent steps were performed as the tier 1 assay. A test sample was tier 2 positive if the percent reduction of ECL signal is greater than the fixed cut point. If a sample was positive in the tier 2 assay, tier 3 testing was performed on the sample. As use of ART can diminish the sensitivity and specificity of an HIV neutralization assay for ADA detection, a binding inhibition assay to HIV trimer, expressing the CD4 binding site and V3 loop (the binding sites of VRC07-523LS and 10-1074, respectively was performed. Serially diluted sample or control were incubated with bNAb coated MSD plate, followed by detection using biotinylated HIV trimer in a sandwich MSD assay format. A reduction of binding activity of bNAb to HIV trimer in sample as normalized to control binding demonstrates if ADA was present in the sample that affects the ability of the bNAb to recognize its target, and therefore, reduce its binding (and potentially neutralizing) capacity. The ECL signals of the samples are plotted on a 5-parameter non-linear regression curve plot where the EC50 (50% binding reduction titer) value is interpolated. Any sample with EC50 titer greater than the sample minimum dilution is considered to be tier 3 positive, and therefore, the ADA is sufficient to impair binding of free drug to target HIV trimer epitope.
